# 5-Aminolevulinic Acid and 24-Epibrassinolide Improve the Drought Stress Resilience and Productivity of Banana Plants

**DOI:** 10.3390/plants11060743

**Published:** 2022-03-10

**Authors:** Mohamed N. Helaly, Hanan M. El-Hoseiny, Nabil I. Elsheery, Hazem M. Kalaji, Sergio de los Santos-Villalobos, Jacek Wróbel, Islam F. Hassan, Maybelle S. Gaballah, Lamyaa A. Abdelrhman, Amany M. Mira, Shamel M. Alam-Eldein

**Affiliations:** 1 Agricultural Botany Department, Faculty of Agriculture, Mansoura University, Mansoura 35516, Egypt; dr.mnehelali@yahoo.com; 2 Horticulture Department, Faculty of Desert and Environmental Agriculture, Matrouh University, Fouka 51511, Egypt; hananelhosieny@yahoo.com; 3 Agricultural Botany Department, Faculty of Agriculture, Tanta University, Tanta 31527, Egypt; nshery@agr.tanta.edu.eg; 4 Department of Plant Physiology, Institute of Biology, Warsaw University of Life Sciences SGGW, 02-776 Warsaw, Poland; hazem_kalaji@sggw.edu.pl or; 5 Institute of Technology and Life Sciences, National Research Institute, Falenty, Al.Hrabska 3, 05-090 Pruszków, Poland; 6 Technological Institute of Sonora, 818 South Ferbero 5th Street, Ciudad Obregon 85000, Mexico; sergio.delossantos@itson.edu.mx; 7 Department of Bioengineering, West Pomeranian University of Technology, 71-434 Szczecin, Poland; jacek.wrobel@zut.edu.pl; 8 Water Relations and Field Irrigation Department, Agricultural and Biological Research Institute, National Research Center, Giza 12622, Egypt; if.hassan@nrc.sci.eg (I.F.H.); msgaballa54@yahoo.com (M.S.G.); 9 Soil, Water and Environment Research Institute (SWERI), Agricultural Research Center, Giza 12619, Egypt; lamyagad91@yahoo.com; 10 Department of Horticulture, Faculty of Agriculture, Tanta University, Tanta 31527, Egypt; amanymira@agr.tanta.edu.eg

**Keywords:** drought, 5-aminolevulinic acid, brassinosteroids, chloroplast degeneration, antioxidants, malondialdehyde, photosynthesis

## Abstract

Plant growth, development, and productivity are adversely affected under drought conditions. Previous findings indicated that 5-aminolevulinic acid (ALA) and 24-epibrassinolide (EBL) play an important role in the plant response to adverse environmental conditions. This study demonstrated the role of ALA and EBL on oxidative stress and photosynthetic capacity of drought-stressed ‘Williams’ banana grown under the Egyptian semi-arid conditions. Exogenous application of either ALA or EBL at concentrations of 15, 30, and 45 mg·L^−1^ significantly restored plant photosynthetic activity and increased productivity under reduced irrigation; this was equivalent to 75% of the plant’s total water requirements. Both compounds significantly reduced drought-induced oxidative damages by increasing antioxidant enzyme activities (superoxide dismutase ‘SOD’, catalase ‘CAT’, and peroxidase ‘POD’) and preserving chloroplast structure. Lipid peroxidation, electrolyte loss and free non-radical H_2_O_2_ formation in the chloroplast were noticeably reduced compared to the control, but chlorophyll content and photosynthetic oxygen evolution were increased. Nutrient uptake, auxin and cytokinin levels were also improved with the reduced abscisic acid levels. The results indicated that ALA and EBL could reduce the accumulation of reactive oxygen species and maintain the stability of the chloroplast membrane structure under drought stress. This study suggests that the use of ALA or EBL at 30 mg·L^−1^ can promote the growth, productivity and fruit quality of drought-stressed banana plants.

## 1. Introduction

Bananas, *Musa* spp., family Musaceae, order Zingiberales, originated in the humid tropics of Southeast Asia and the South Pacific around 8000–5000 BC and are believed to be the world’s first cultivated fruit crop. They are one of the most important fruit crops and were adapted from the humid tropics to the vast subtropical and Mediterranean climates in many countries; hence, the banana is also called the “Queen of Tropical Fruits”. The fruits were brought to the west by Arab conquerors and then carried to the New World with explorers and missionaries. The name “banana” came from the Arabic word “banan”, meaning “finger.” The modern seedless bananas (3N), referring to banana (dessert bananas) and plantain (cooking bananas), are descended from the wild seeded species (2N) *M. acuminata* and *M. balbisiana*. Dessert bananas usually refer to the soft and sweet fruits, especially those belonging to the Cavendish subgroup, which are more suitable for international trade than any other varieties. In some of the least-developed and low-income food-deficit countries, bananas are a staple food source and serve as a cash crop for income generation (i.e., they account for 75% of the total monthly household income for smallholder farmers) [1,2,3].

Bananas are grown in more than 130 countries around the world, with a total cultivated area of about 5,158,582 hectares and a total production of 116,781,658 tons. The top ten producers are India, China, Indonesia, Brazil, Ecuador, Philippines, Guatemala, Angola, Tanzania, and Colombia. The climate of Egypt is suitable for banana cultivation. The total cultivated area in Egypt is about 30,389 hectares, with a total annual production of 1,359,297 tons in 2019 [4]. Within a group of 98 producing countries, Egypt ranked 18th in 2020 [5]. Banana production is the fifth largest fruit crop in Egypt after citrus, mangoes, olives, and grapes. The most commonly grown varieties are Williams, Grandnain, and Maghrabi [6]. Bananas are considered a good source of energy with an estimated favorable wholesale price of USD 0.69 per kg in 2021 [7].

Bananas are among the water-loving plants that require over 25 mm of rainfall per week, which is equivalent to 1300 mm per year. More than two-thirds of bananas grown for export in the world are irrigated [8]. The plants can be grown successfully in all irrigated semi-arid regions of the world and tolerate a short period of water deficit [9]. In traditional banana-growing areas, prolonged drought is not common, although it is a potential abiotic stress as a short dry spell. Inherent plant-related problems, such as a long crop duration (9–11 months), high leaf area index, and shallow root system, make drought a potential threat to bananas [1]. Water loss through transpiration can reach up to 30–63 m^3^·ha^−1^·day^−1^, depending on the radiation, wind, and humidity [10]. Therefore, the plants require adequate irrigation during dry periods to avoid a reduction in fruit yield and quality [11].

Drought is a major problem for global agriculture, permanently affecting 28% of the world’s soils [12], especially in the tropical and subtropical regions [9]. It refers to a lack of water in the root zone, which affects plant growth and productivity [13]. Water deficiency suppresses cell division and expansion, causes membrane injury, and reduces root differentiation, shoot length, leaf area, leaf water potential, photosynthetic rate, nutrient uptake, soluble protein content, and the activity of metabolic enzymes, especially those associated with adenosine triphosphate (ATP) synthesis [14,15]. Drought stress also increases the formation of reactive oxygen species (ROS) and reactive nitrogen species (RNS), which disrupt the redox regulatory function of the cells [16]. The damage caused by water stress depends on plant genotype-specific traits, growth stage, stress intensity and duration, and other environmental conditions [17,18,19]. Plant tolerance to drought can be through drought avoidance and/or dehydration tolerance, which is ultimately measured by plant reproductive success [20]. Drought avoidance involves morpho-anatomical strategies, such as deep rooting and conservative water use [21]. Dehydration tolerance involves the ability to endure partial dehydration but to remain viable and resume growth when water is available [10]. Strategies of plant survival under drought stress also include physiological responses, such as rapid stomatal closure and efficient water use, and biochemical responses, such as the synthesis of osmolytes, aquaporins, and a strong antioxidant system to maintain cell homeostasis [22,23]. 

Water scarcity is becoming a current problem in Egypt, as water resources and rainfall are limited, and the Nile River is the main water resource [24]. This may become a limiting factor of the Egyptian banana industry in the future. Under such conditions, there is a need to reduce agricultural water demand and increase the economic productivity of water through improved on-farm management with advanced irrigation technologies and schedules [25]. Research has been conducted to reduce water use through deficit irrigation (DI) [14,26,27,28,29,30], avoiding growth stages that are sensitive to water shortages, such as flowering and early fruit growth [31]. Deficit irrigation is an irrigation strategy in which the amount of water applied is less than the full water requirement (WR) of a crop to develop a desirable stress that has a minimal impact on plant growth and productivity [32]. To compensate for the reduction of WR, the foliar application of various plant hormones or growth regulators is a recognized strategy to alleviate the negative effects of water deficit by regulating plant metabolism and biochemical processes, such as chlorophyll synthesis, photosynthesis, nutrient uptake, protein synthesis, and antioxidant metabolism, which are directly or indirectly involved in the stress tolerance mechanism, thus improving plant growth, development, productivity, and fruit quality [33].

The potential plant growth regulator 5-aminolevulinic acid (ALA) is a type of non-protein amino acid that occurs naturally in plants, animals, fungi, and bacteria [34] at very low concentrations (60 μM) [35]. It has a molecular weight (MW) of 167.59 g·mol^−1^ and is a white to off-white odorless crystalline solid that is very soluble in water, with the chemical name 5-amino-4-oxy-pentanoic acid hydrochloride (C_5_H_9_NO_3_–HCl) [36]. At very low concentrations, it affects plant growth and development, but higher concentrations promote the production of ROS and, thus, oxidative stress metabolism in the plant [37]. It is an important precursor in the biosynthesis of all porphyrins, such as chlorophyll, heme, vitamin B12 and phytohormones. It has great potential in agriculture as a growth-promoting factor, as it is a non-toxic endogenous substance involved in plant growth and productivity under various abiotic stresses [33,38,39]. Exogenous application of ALA enhances plant defense mechanisms against cold [40], salinity [41], and drought stress [42,43] and increases chlorophyll content, photosynthetic rate, antioxidant enzymatic capacity, and nutrient contents, which improves plant growth and overall yield [38]. The application of ALA improved turf quality and induced drought tolerance in Kentucky bluegrass by reducing oxidative damage with increased non-enzymatic and enzymatic antioxidant capacity at the transcriptional and post-transcriptional levels [44].

In addition to the five main groups of plant phytohormones; auxin, gibberellins, cytokinins, ABA, and ethylene, brassinosteroids (BRs) are considered to be a sixth group of phytohormones of isoprenoid origin, which are of great importance in protecting plants against environmental stress [45,46,47]. They are more than 70 structurally and functionally related steroids considered to be phytohormones. Among them, both 24-epibrassinolide (EBL) (C_28_H_48_O_6_, 480.7 g·mol^−1^ MW) and 28-Homobrassinolide (C_29_H_50_O_6_, 494.7 g·mol^−1^ MW) are the most bioactive BRs [48]. Brassinosteroids have pleiotropic effects that affect various physiological processes, such as plant growth and development, rhizogenesis, seed germination, photosynthesis, enzymatic activity, senescence, and abiotic and biotic stress tolerance [49,50,51] at very low concentrations [48,52]. They delay senescence by reducing ethylene evolution and the respiration rate [53]. They also improve the photosynthetic apparatus and increase the plant’s ability to capture sunlight, which affects the biosynthesis of pigments and increases photosynthetic efficiency [54], especially under drought stress [55]. Exogenous application of BRs improved plant water use efficiency and nitrogen uptake, and stimulated some antioxidant enzymes, such as superoxide dismutase (SOD), catalase (CAT), and peroxidase (POD) [54,56].

Previous findings on the use of ALA or EBL to improve plant tolerance to different types of environmental stresses have focused on low-light stress [57], UV stress [58], salt stress [41,59,60,61], heat stress [62], cold stress [57,59,63], and drought stress [42,64] in other crops but not in bananas. Very few reports focused on the use of ALA to improve the growth of drought-stressed banana seedlings without estimating plant productivity [43] and EBL to improve the heat stress tolerance of in vitro banana seedlings [65]. To date, there is no report on the use of ALA or EBL to improve the mechanism of drought tolerance and productivity of banana plants. Therefore, this study is considered as the first in this regard.

## 2. Results

### 2.1. The Application of ALA or EBL Increased the NPK Content under Drought 

**Figure 1:**
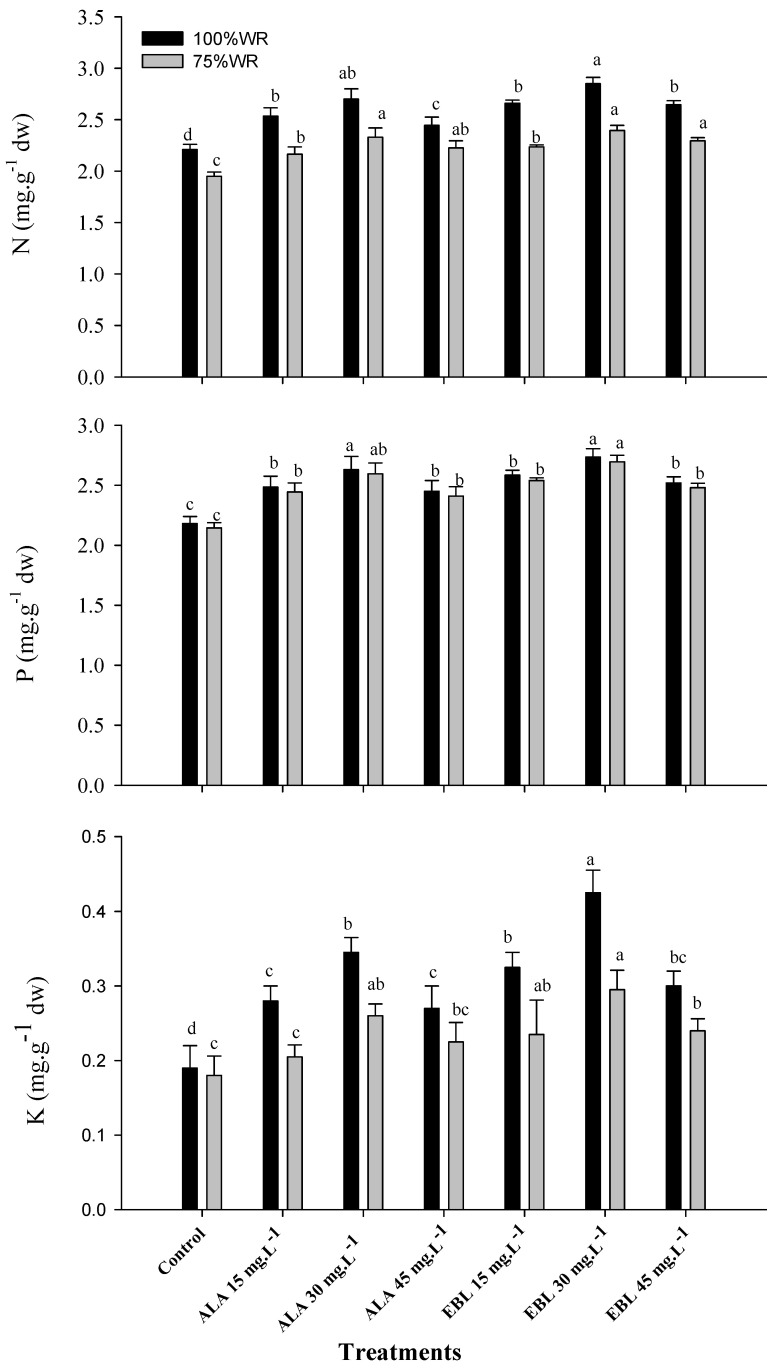
Effect of foliar spray with 5-aminolevulinic acid (ALA) or 24-Epibrassinolide (EBL) on the leaf nitrogen (N), phosphorus (P), and potassium (K) contents of drought-stressed “Williams” banana plants during the 2018/2019 and 2019/2020 seasons (*n* = 5). The results are the averages of both seasons, and Duncan’s multiple range test (DMRT) was used for mean comparisons of treatments under each water requirement (WR) level (*p* ≤ 0.05).

Leaf analysis has shown that reducing the water requirements of ‘Williams’ banana to 75% significantly decreased nitrogen (N) and potassium (K) uptake compared to the control, while the effect on phosphorus (P) was insignificant in both seasons (Figure 1). The application of ALA or EBL significantly improved the NPK content in the leaf of the stressed plants. The application of EBL was more effective in this regard and the most pronounced effect was recorded with the application of EBL (30 mg·L^−1^), followed by ALA (30 mg·L^−1^), although the difference was insignificant between both treatments. The effect of ALA on the K content was insignificant at the lowest and highest concentrations in comparison to the control. It was also found that increasing the concentrations of ALA or EBL up to 30 mg·L^−1^, either alone or in combination with 75% WR, increased the NPK content of the leaves. Any further increase in the concentration of ALA or EBL decreased the NPK content in both seasons. In this regard, the only significant difference was noticed with EBL (45 mg·L^−1^) affecting both the P and K contents in stressed plants. The increased NPK content was also associated with an increased content of amino acids and total proteins (data not shown).

### 2.2. The Application of ALA or EBL Suppressed Chloroplast Degeneration under Drought 

**Figure 2:**
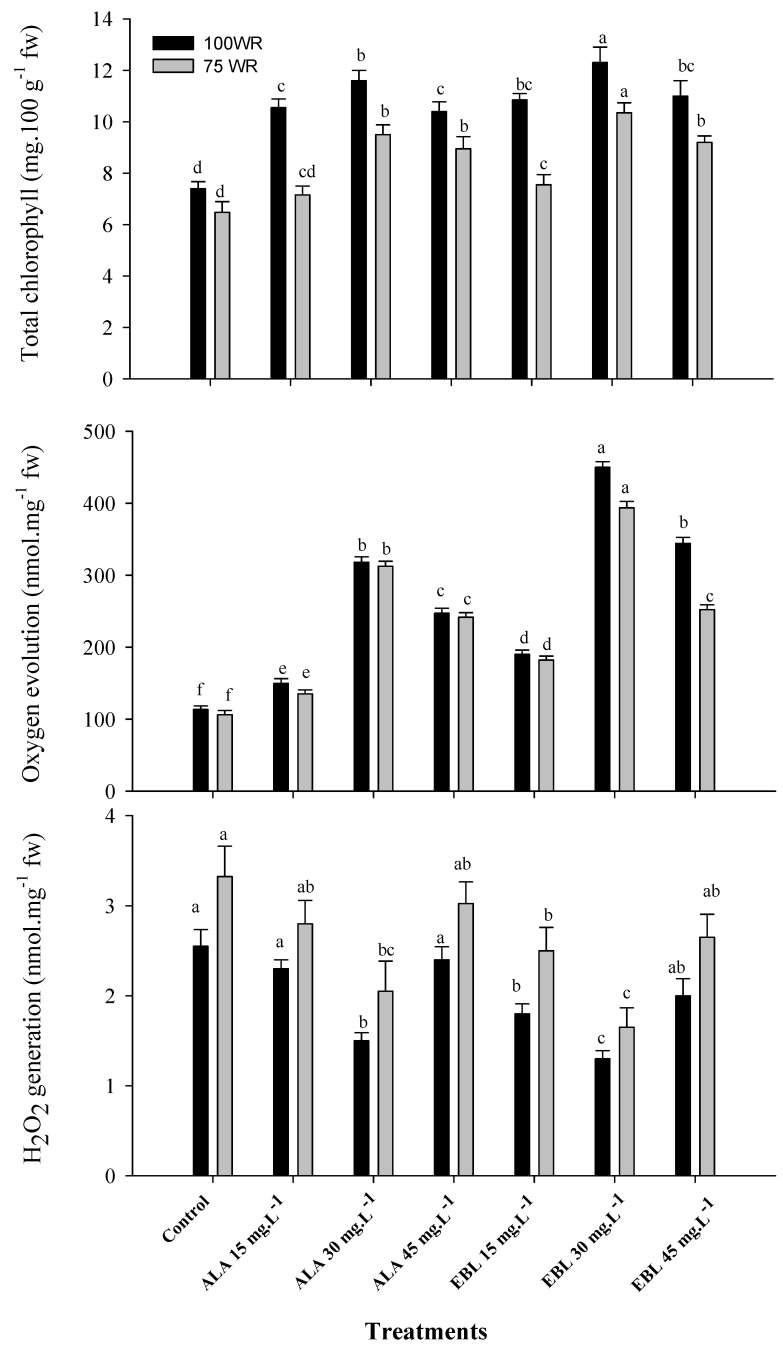
Effect of foliar spray with ALA or EBL on the leaf total chlorophyll content, photosynthetic oxygen evolution, and H_2_O_2_ generation of drought-stressed “Williams” banana plants during the 2018/2019 and 2019/2020 seasons (*n* = 5). The results are the averages of both seasons, and DMRT was used for mean comparisons of treatments under each WR level (*p* ≤ 0.05).

As a result of the water stress conditions, chloroplast degeneration was represented by a reduction in total chlorophyll content and photosynthetic oxygen evolution, which was associated with an increase in free nonradical H_2_O_2_ generation, as shown in Figure 2. Foliar spray of ALA or EBL significantly enhanced the chlorophyll content and photosynthetic oxygen evolution and decreased H_2_O_2_ generation compared to the control in both seasons. The chlorophyll content and the rate of photosynthetic oxygen evolution in chloroplasts significantly increased with elevated concentrations of ALA or EBL up to 30 mg·L^−1^ and decreased thereafter, although their values were still higher at the highest concentrations (45 mg·L^−1^) than at the lowest (15 mg·L^−1^). Conversely, the level of H_2_O_2_ generation decreased with elevated concentrations of either ALA or EBL up to 30 mg·L^−1^ and increased thereafter to reach a level higher than that at the lowest concentration, although the difference was insignificant. In all cases, including H_2_O_2_ generation, the application of EBL was more effective than that of ALA, and the most pronounced effect was recorded with EBL (30 mg·L^−1^), followed by ALA (30 mg·L^−1^), compared to the control in both seasons.

### 2.3. The Application of ALA or EBL Maintained Membrane Integrity under Drought 

The stress conditions affected the membrane stability of the chloroplast due to the increased level of lipid peroxidation, which was represented by the increase in the content of malondialdehyde (MDA) and the percentage of electrolyte leakage (EL) during both seasons (Table 1). The application of ALA or EBL generally reduced lipid peroxidation, and EBL was more effective in this regard. The application of EBL (30 mg·L^−1^) was the most effective on the MDA content and EL percentage in both seasons. The application of ALA (30 mg·L^−1^) was the second most effective on MDA content during the first season, but the difference was insignificant compared to EBL (15 mg·L^−1^) during the second season. The application of EBL (15 mg·L^−1^) was the second most effective on the EL percentage in both seasons. However, the difference was insignificant compared to ALA (30 mg·L^−1^) during the first season. 

### 2.4. The Application of ALA or EBL Activated the Antioxidant Enzyme System under Drought 

**Figure 3:**
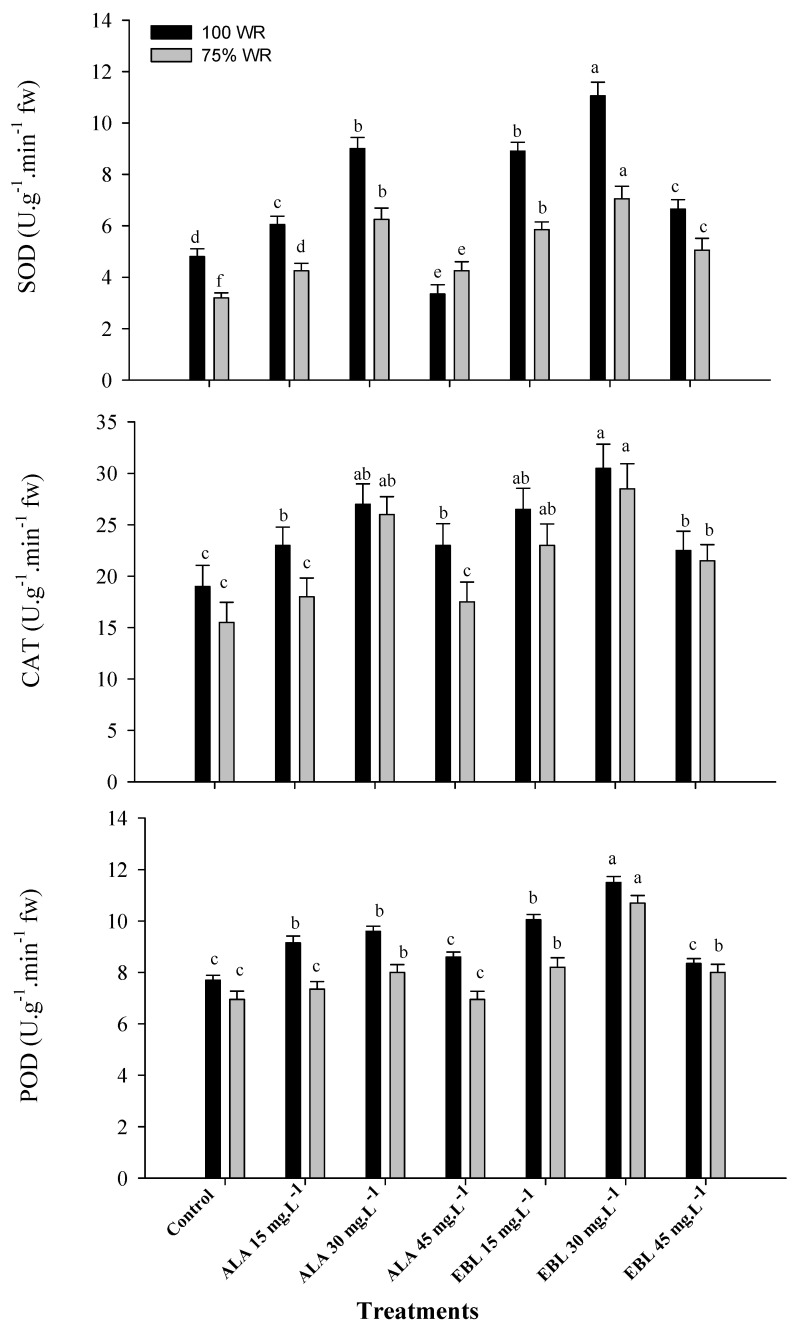
Effect of foliar spray with ALA or EBL on the activity of superoxide dismutase (SOD), catalase (CAT), and peroxidase (POD) in drought-stressed “Williams” banana plants during the 2018/2019 and 2019/2020 seasons (*n* = 5). The results are the averages of both seasons, and DMRT was used for mean comparisons of treatments under each WR level (*p* ≤ 0.05).

Drought stress significantly affected the antioxidant enzyme capacity, represented by a significant reduction in the activity of SOD, CAT, and POD compared to well-watered ‘Williams’ banana during both seasons (Figure 3). The foliar application of ALA or EBL positively affected the activity of these enzymes in both seasons, except for the activity of CAT and POD, which was not affected by ALA (15 or 45 mg·L^−1^) compared to the control in both seasons. It was also found that enzyme activities significantly increased when the concentration of ALA or EBL was increased up to 30 mg·L^−1^ and decreased thereafter. The application of EBL was more effective compared to ALA, and EBL (30 mg·L^−1^) was the most effective, followed by ALA (30 mg·L^−1^), which was not significantly different compared to EBL (15 mg·L^−1^) affecting SOD activity. The application of ALA (30 mg·L^−1^) on CAT and POD activity was also insignificant compared to EBL (15 and 45 mg·L^−1^). In general, the application of EBL (45 mg·L^−1^) showed a better effect compared to ALA (15 and 45 mg·L^−1^) in both seasons, confirming the effective role of EBL compared to ALA. 

### 2.5. The Application of ALA or EBL Altered Phytohormone Levels under Drought

Drought stress also decreased the levels of auxin (IAA) and cytokinins (CKs) but increased those of abscisic acid (ABA) compared with the control. The application of ALA or EBL significantly improved IAA and CKs contents but decreased ABA content in stressed plants during both seasons (Table 2). The results also showed that IAA and CK levels significantly increased with the increased concentrations of ALA or EBL up to 30 mg·L^−1^ but decreased thereafter, although their concentrations at 45 mg·L^−1^ were still higher than that at the lower concentration (15 mg·L^−1^), except for the similar CKs concentration with ALA application in the first season. The levels of ABA also decreased with the application of ALA or EBL up to 30 mg·L^−1^ and then increased at 45 mg·L^−1^. The strongest effect in this regard was found when EBL (30 mg·L^−1^) was applied, followed by a similar effect of both ALA (30 mg·L^−1^) and EBL (45 mg·L^−1^) on IAA and ABA in both seasons and on CKs during the first season. The application of ALA (30 mg·L^−1^) was more effective than EBL (45 mg·L^−1^) on CK activity during the second season.

### 2.6. The Application of ALA or EBL Improved Yield and Fruit Quality under Drought 

Yield parameters, represented by bunch weight, number of hands per bunch, and number of fruits (fingers) per hand, were reduced by drought stress conditions in both seasons. The application of ALA or EBL significantly improved the yield parameters of both stressed and non-stressed plants (Table 3). Under drought conditions, the yield parameters increased significantly with increased concentrations of ALA or EBL up to 30 mg·L^−1^, except for bunch weight during the first season. The most pronounced effect was recorded with EBL at 30 mg·L^−1^. The application of ALA (45 mg·L^−1^) or EBL (15 mg·L^−1^) combined with 75% WR positively improved bunch weight compared to non-stressed plants receiving the same treatments during both seasons. On the other hand, the number of fruits per hand was also improved with the application of ALA (45 mg·L^−1^) during the second season only. In the stressed ‘Williams’ plants, the number of fruits per hand was still higher at the highest concentration (45 mg·L^−1^) than that at the lowest concentration (15 mg·L^−1^) of ALA during both seasons. 

### 2.7. The Application of ALA or EBL Enhanced Fruit Physical Characteristics under Drought

**Figure 4:**
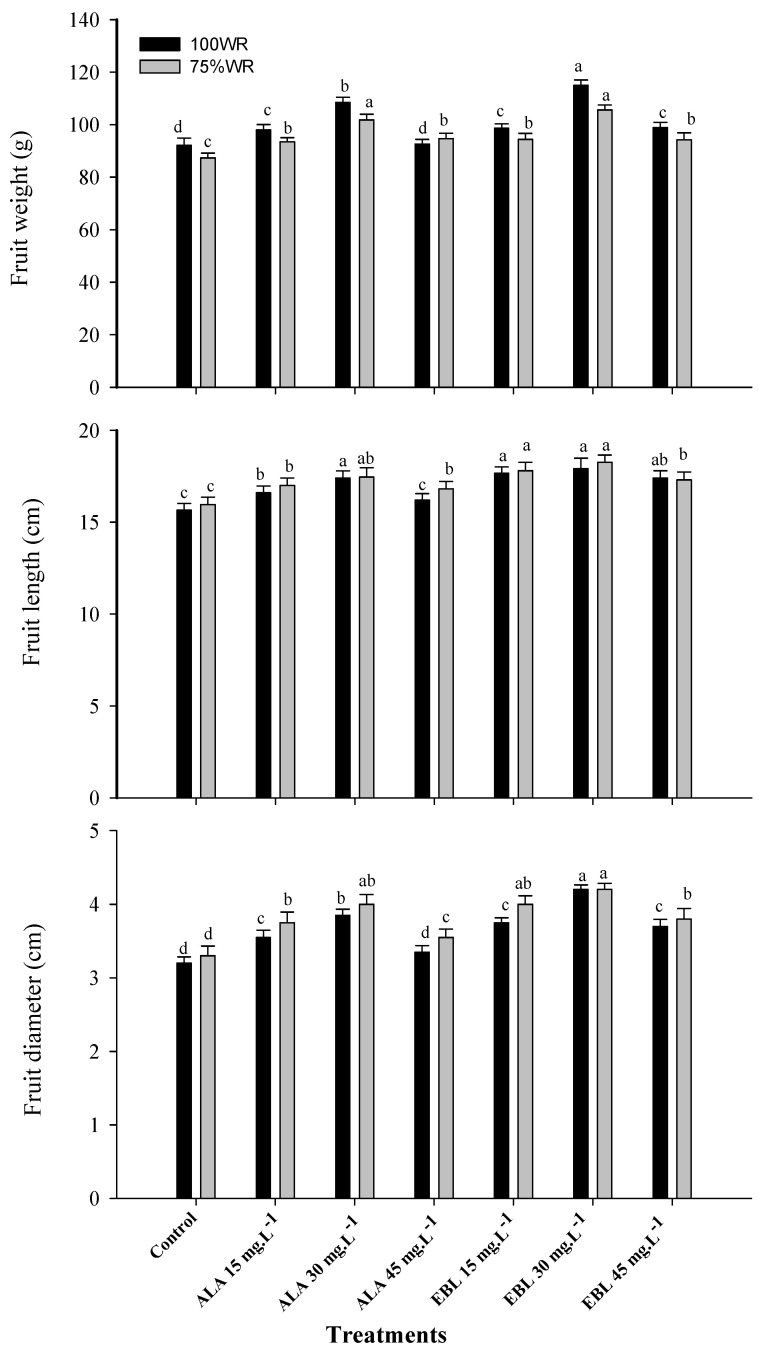
Effect of foliar spray with ALA or EBL on the fruit weight, length, and diameter of drought-stressed “Williams” banana plants during the 2018/2019 and 2019/2020 seasons (*n* = 5). The results are the averages of both seasons, and DMRT was used for mean comparisons of treatments under each WR level (*p* ≤ 0.05).

Under drought stress conditions, fruit weight decreased in both seasons, whereas fruit length and diameter showed a slight increase (Figure 4). Stressed banana plants treated with either ALA or EBL showed significant improvement in all fruit physical characteristics compared to the control, with the highest values recorded for EBL (30 mg·L^−1^), although the difference was insignificant compared to ALA (30 mg·L^−1^). The results also showed an improvement in the fruit weight of stressed plants treated with ALA (45 mg·L^−1^) as compared to the non-stressed treated ones. Similarly, the fruit length and diameter of stressed plants treated with most concentrations of ALA or EBL were improved compared to the non-stressed treated plants.

### 2.8. The Application of ALA or EBL Enhanced Fruit Chemical Characteristics under Drought

**Figure 5:**
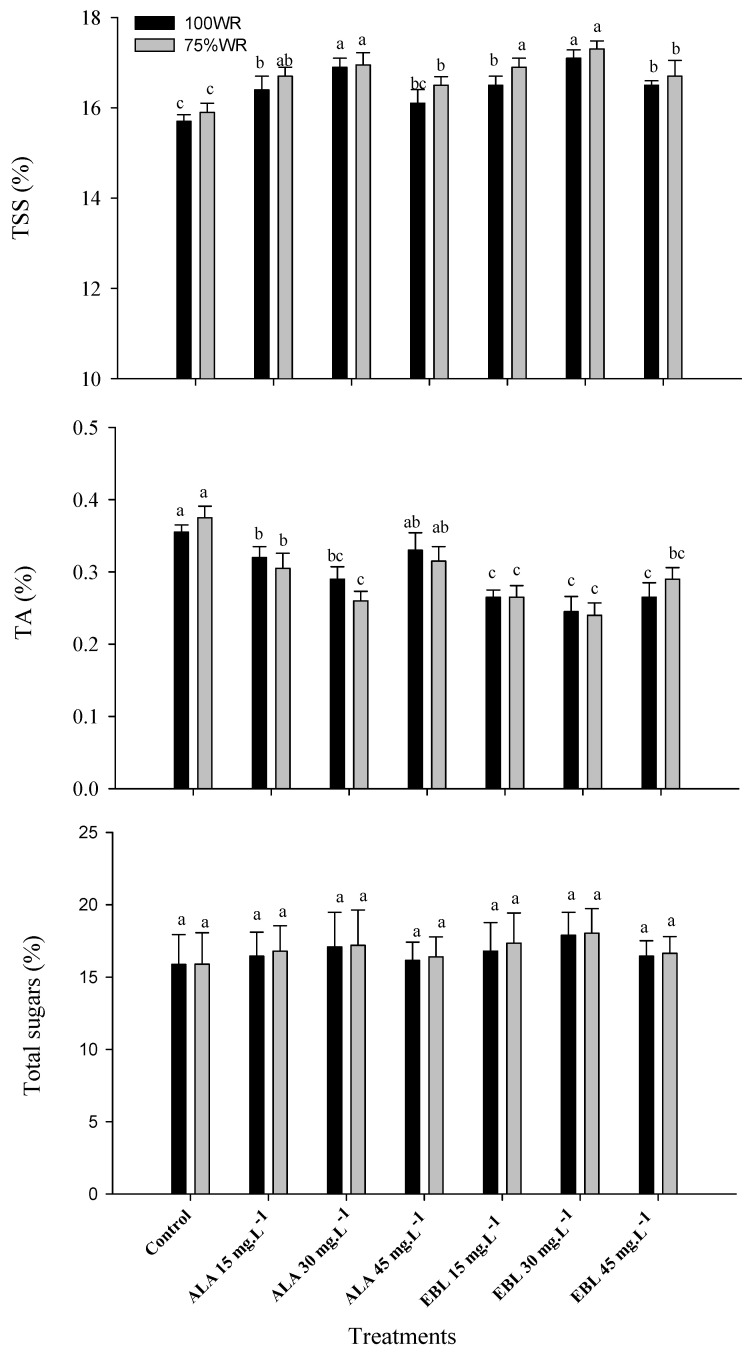
Effect of foliar spray with ALA or EBL on the percentage of total soluble solids (TSS), total acidity (TA) and total sugars in the fruit of drought-stressed “Williams” banana plants during the 2018/2019 and 2019/2020 seasons (*n* = 5). The results are the averages of both seasons, and DMRT was used for mean comparisons of treatments under each WR level (*p* ≤ 0.05).

Compared to the control, fruit chemical properties such as total soluble solids (TSS) and total acidity (TA) slightly increased in stressed plants, while total sugars were almost the same during both seasons (Figure 5). The application of either ALA or EBL on both non-stressed and stressed plants improved the TSS and decreased TA, while total sugars remained almost the same compared to the control. The application of EBL (30 mg·L^−1^) significantly improved the TSS of stressed plants compared to the control and the highest concentrations of either ALA or EBL. The same treatment significantly reduced the TA in comparison to the control, as well as the highest and the lowest concentrations of ALA. Despite EBL (30 mg·L^−1^) being the most effective treatment on total sugar content, it was not significantly different from all other treatments and the control.

## 3. Discussion

The banana is classified as a water-loving plant [8]. Therefore, the plant is sensitive to soil moisture stress, which severely affects its growth and productivity [14]. As water scarcity has recently become a global problem [66], water deficit regimes have been used to reduce agricultural water demand and increase economic productivity [14,25,26,27,28,29,30]. Meanwhile, the foliar application of plant hormones/regulators can play an important role in inducing the plant’s ability to withstand adverse stress conditions, thereby improving plant growth, productivity and fruit quality [33]. In general, the results of this study showed the negative effects of drought stress on the growth and productivity of ‘Williams’ banana. The results also addressed the role of foliarly sprayed ALA or EBL in mitigating the negative effects of drought by improving nutrient uptake (Figure 1), maintenance of chloroplast integrity (Figure 2 and Table 1), antioxidant capacity (Figure 3 and Table 2), and, ultimately, plant productivity (Table 3) and fruit quality (Figure 4 and Figure 5). It could also be mentioned that according to the previous reports [38,49], the small used concentrations of either ALA or EBL were effective, with the best results at 30 mg·L^−1^ for each. In general, EBL was more effective compared to ALA.

Drought stress negatively affects cell osmotic pressure, hormonal balance, nutrient uptake and accumulation, water relations, and the antioxidant defense system [67]. Stressed banana plants showed an overall reduction in plant growth due to reduced stomatal conductance, leaf size, and photosynthetic rate [68]. Prolonged drought can also induce leaf aging and senescence processes [69]. Banana plants rarely reach their full genetic productivity potential because photosynthesis is limited by water stress [14]. Expanding tissues, such as developing leaves and growing fruit, are among the first to be affected. When the soil begins to dry, stomata close and leaves remain highly hydrated, probably due to root pressure, but productivity is impaired [70]. Water deficit also induces the disintegration of the thylakoid membranes of chloroplasts; this leads to chlorophyll degradation due to increased chlorophyllase activity, which significantly inhibits photosynthesis [71]. Chlorophyll content is used as an indicator of chloroplast development and photosynthetic activity [72], as it is very sensitive to abiotic stress [48], as shown in the results (Figure 2). Photosynthetic oxygen evolution, indicative of gross photosynthesis, was also affected by drought stress (Figure 2). Photosystem II (PSII) catalyzes light-driven electron transfer from water, an electron source unique to oxygenic photosynthesis, in the luminal side of the PSII complex. This PSII core complex consists of 16 proteins; 13 are membrane internal, and 3 are located on the luminal side of the membrane, stabilizing the oxygen-evolving complex [73]. These proteins could be affected by drought stress.

Drought often leads to the formation of ROS, which are highly toxic and can react with proteins, lipids, and DNA, accelerating the aging process of chloroplasts, thus reducing photosynthetic capacity and decreasing plant growth and productivity [74]. Abiotic environmental stresses affect the plant through osmotic stress. Cell homeostasis is maintained against osmotic stress by the mechanism of osmotic adjustment, which is a primary stress-adaptive motor that positively correlates with plant production under drought conditions in various crops [75]. The mechanism of osmotic adjustment leads to the synthesis of organic osmolytes (e.g., sugars and proline) [76], non-enzymatic antioxidants (e.g., ABA, ascorbate, and glutathione), and enzymatic (scavenger enzymes) antioxidants (e.g., SOD, CAT, and POD) [77] to balance the osmotic pressure of the cytosol and vacuole with that of the external environment [78]. The enzymatic components of the antioxidant system include SOD, the most effective intercellular metalloenzyme [79], which plays a fundamental role in catalyzing the dismutation of superoxide anions (O^−^_2_^.^) to dioxygen (O_2_), and hydrogen peroxide (H_2_O_2_), which increased under drought conditions, as shown in Figure 2. The reduction in SOD activity indicates that the antioxidant defense system is slightly impaired [80]. As a primary antioxidant enzyme, CAT was first discovered and characterized. It, together with ascorbate peroxidase (APX) and POD, plays an important role in ROS detoxification under stress by converting H_2_O_2_ to water (H_2_O) and singlet oxygen (^1^O_2_) [81].

The formation of ROS, as one of the aging/senescence mechanisms of the organism [74,82], is also responsible for the stress-dependent peroxidation of membrane lipids and the formation of MDA (Table 1) as the end product of peroxidation; this represents the degree of membrane damage under stress conditions [83]. Electrolyte leakage (Table 1) is also used as another indicator of drought stress, which represents higher electrical conductivity due to low cytoplasmic membrane stability [84]. The reasons for cell wall damage and membrane instability under drought conditions are actually related to stomatal closure, which leads to a reduction in CO_2_ fixation, while the light-dependent reactions of photosynthesis and electron transport continue at normal rates. Therefore, there is a limited amount of nicotinamide adenine dinucleotide phosphate (NADP) to accept electrons. Therefore, oxygen acts as an electron receptor, and this alternative reaction may lead to the accumulation of toxic oxygen compounds, such as O^−^_2_^.^, H_2_O_2_, hydroxyl radicals (OH), and ^1^O_2_ [85], thus promoting cellular aging and senescence [86,87,88]. Superoxide dismutase (SOD) represents the first line of defense against lipid peroxidation and maintains membrane stability in stressed cells [81]. The up-regulation of the enzymes SOD, CAT, and POD under drought stress suggests the presence of a potential system in plant cells to cope with stress [89]. This is actually in contradiction with the current results showing a decrease in SOD, CAT, and POD under drought conditions (Figure 3), although some other non-enzymatic antioxidants, such as ABA, increased (Table 2). This could be justified by the fact that the antioxidant defense system is little affected [80], since banana is not drought tolerant [8]. 

The application of ALA has shown a positive effect on the regulation of various metabolic processes [90] and the promotion of plant growth, chlorophyll content, photosynthetic rate, stomatal conductance, leaf N, P, K, Ca, Mg, and Mn content, and sugar content of non-stressed [91] and stressed plants [43,44]. It has been reported that ALA promotes plant growth and crop yield by enhancing chlorophyll biosynthesis, C fixation, and N assimilation under stressful conditions [34,92]. It is well documented that chlorophyll synthesis depends primarily on enzymes involved in the synthesis and degradation of ALA [93]. 5-aminolevulinic acid plays an important role in the synthesis of the chlorophyll molecule via the monovinyl and divinyl monocarboxylic acid pathway [94] or via another ALA pathway in which the carbon skeleton of glutamate is integrated into the first step of chlorophyll biosynthesis through the formation of protochlorophyllide [95]. The exogenous application of ALA induced plant stress tolerance and growth by increasing the accumulation of K and maintaining a high K/Na ratio in the roots [96], resulting in an improved plant nutrient status, altered light response, enhanced photosynthetic assimilation, and improved interception of ROS [97]. The increase in the Mg and Mn content is important for the Mg and Mn clusters of PSII [73]. Membrane damage and ROS production are multiple negative effects of salinity and drought stress [20]. Thus, the attenuation of drought effects in banana plants treated with ALA could be due to increased membrane stability (Table 1) by reducing the activities of phospholipase D (PLD) and lipoxygenase (LOX), resulting in increased activity of the antioxidant system [42]. The application of ALA also reduced electrolyte loss under cold stress conditions [37]. Plants under drought stress treated with ALA showed a significant improvement in non-enzymatic antioxidants such as anthocyanins, carotenoids, tocopherols, phenols, glucosinolates, flavonoids and ascorbic acid [98], and enzymatic antioxidants such as SOD, CAT [99] and POD [100], as shown in Figure 3, and, hence, ROS-induced stress was alleviated [101]. This could be the reason for the increased chlorophyll content (Figure 2) and total yield (Table 3) compared to the control, as shown in barley [102] and wheat [103]. This could also indicate that ALA stabilized the integrity of the chloroplast structure and delayed its degradation under drought stress conditions [104]. It was also reported that ALA upregulated the expressional level of the genes encoding ribulose-1,5-biphosphate carboxylase/oxygenase (RuBisCo) (i.e., *BnRBCL* and *BnRBCS*) and other genes, such as *BnUGT79B1*, *BnMYB12,* and *MYB28,* that regulate the synthesis of phenolic compounds and glucosinolates, respectively [105]. 

The application of ALA also improved the total and reducing sugars, fruit weight and volume, and flesh percentage in date palm [106]. This improvement in plant growth and productivity is generally associated with all pathways involved in root growth (particularly the mechanism of auxin transport and biosynthesis) [107], chlorophyll biosynthesis, and total chlorophyll content [33], which are associated with improved yield and fruit quality (Table 3, and Figure 4 and Figure 5) due to an increased photosynthetic rate [14]. It was reported that ALA inhibits ABA-induced stomatal closure via the reduction of the H_2_O_2_ and Ca^2+^ levels in guard cells and simultaneously improves the photosynthesis of stressed plants [108]. Cytokinins and auxins were also reported to induce stomatal opening by decreasing H_2_O_2_ levels in guard cells [109]. 5-aminolevulinic acid significantly enhanced the expression of *YUC2* [108], one of the key genes of auxin biosynthesis [110]. It also improves the PIN-mediated regulation of auxin transport and distribution, which controls cell division and elongation in the root apex [111], two major processes determining root growth. The PIN proteins, the auxin efflux carriers, play crucial roles in regulating the direction of auxin flow. Thus, they are very important for auxin-regulated root growth. Both PIN1 and PIN7 mainly regulate acropetal auxin transport, while PIN2 specifically influences the basipetal auxin transport [111,112]. An et al. [107] reported that ALA regulates PIN proteins at both the transcriptional and post-transcriptional levels and its effect on root growth in a concentration-dependent manner. Low concentrations (e.g., 10 mg·L^−1^) improved root elongation, while higher concentrations (e.g., 20 mg·L^−1^) inhibited it but stimulated the initiation of lateral roots. Improved root growth eventually improves water and nutrient absorption. The results of the present study showed that ALA improved the drought tolerance mechanism of the plant, and this might be related to its effect on improving the nutrient levels such as N, P, K (Figure 1), Ca, and Mg, total sugars, and amino acids, which are the main components of the vacuole-like organelles called “lutoids” [113]. Lutoids are the main component of banana latex, which are actively able to transport ions across membranes and improve the osmotic potential activity of the plant under stress conditions [114]. It has also been reported that ALA suppresses ABA biosynthesis in leaves but promotes it in roots of stressed plants, which is important to promote root growth and improve water uptake and conductivity [115]. Increasing the ABA concentration could increase catechin and malvidin synthesis in moderately water-stressed plants, which can improve fruit color and sugar content and reduce TA [116]. The reduction in TA under deficit irrigation conditions could be due to the reduction in malic acid content associated with reduced water content [117] due to the low availability of the metabolites used to build acids [118], in addition to the use of acids as substrates in respiration [119]. 

Similarly, EBL played an important role in protecting plants against environmental stress [45,46,47]. The foliar spray of EBL regulated the chloroplast ultrastructure, chlorophyll content (Figure 2), photosynthetic capacity, carbohydrate metabolism, and the biosynthesis of IAA, CKs, ABA (Table 2), gibberellins (GAs), jasmonic acid, and various types of endogenous BRs and ultimately improved plant growth and productivity (Table 3) under stressful conditions [120]. It also improved cell division, elongation and differentiation, photomorphogenesis, flower development, pollen sterility, stomatal development, and vascular development, as well as the capacity of the antioxidant system and stress response enzymes, such as SOD, CAT, POD (Figure 3), glutathione reductase (GR), and APX [48,72]. The improved yield (Table 3) and physical fruit characteristics (Figure 4) under drought conditions might be related to the improved cell division, elongation, and flower development under stress conditions. The positive effect of EBL on root cell division and elongation, in addition to improved vascular differentiation, increased the capacity of the root system in absorbing water and nutrients under drought conditions [48,121]. It was reported that EBL induces the signaling pathway, eventually leading to the activation of the brassinazole-resistant 1 (BZR1) transcription factor (TF), a positive regulator of the BRs signaling pathway. The quiescent center (QC) of the root meristem is regulated by the BRs at vascular and organizing center (BRAVO) TF that inhibits QC division [122]. Moreover, EBL can lead to the deactivation of glycogen synthase kinase 3 (GSK3), which results in the activation of BZR1-TF and inhibition of BRAVO, which eventually leads to efficient cell division and root elongation [123]. Likewise, the enlargement of epidermal and mesophyll cells resulted in an enlarged leaf area and an increased photosynthetic capacity and fruit growth under such conditions [124]. This might be related to the positive effect of EBL on the regulation of IAA and CKs levels in the plant [125,126]. The foliar spray of EBL decreased chlorophyll degradation and increased its biosynthesis under stressful conditions [48]. Moreover, EBL regulated the association of the chlorophyll molecule (by regulating chlorophyllase activity) with the membrane protein to maintain the stability of the chloroplast thylakoid membrane [127]. In addition, EBL was reported to mitigate the negative effects of stress conditions and to regulate the plant defense system by regulating the transcription level of defense genes, such as the RuBisCO activase gene, which plays a key role in photosynthesis under drought and heat stress conditions [128]. Furthermore, EBL increased the CO_2_ assimilation rate, the quantum yield of PSII, the RuBisCO activity, and the expression of RuBisCO genes (*rbcL* and *rbcS*) [129], plus it increased the chlorophyll diversity to enhance photosynthetic capacity. 

The application of EBL showed improvement in the expression of some other genes, including those involved in the auxin signaling pathways, such as auxin-induced protein, auxin-responsive protein, and auxin-binding protein (e.g., *Cs453*). Genes involved in cytokinin dehydrogenase (CKX), the enzyme that inactivates CKs, were also downregulated by EBL, leading to a significant role in maintaining the well-organized CKs functions. The protein phosphatase type 2C24 (*P2C24*), identified as a second component of the ABA signaling pathway was also downregulated [130]. Furthermore, EBL increased N, P, K, Ca, Mg, Mn, Cu, and Zn ions, and decreased Na and Cl ions, promoting ion homeostasis in roots and leaves, which ultimately improved plant growth [131]. Phosphorus is a key component of the ATP formed during photosynthesis that supports plant growth and development, stimulates root development, and improves overall crop quality. Potassium also helps regulate water balance in the plant by affecting water uptake by the roots and water loss through transpiration. Therefore, it can improve the plant’s tolerance to drought [55]. In addition, EBL increased the H^+^-ATPase and Ca^+2^-ATPase activities in roots and leaves, which are responsible for establishing an electrochemical potential gradient to maintain the ion balance in the plant, alleviating the stressful conditions [132]. A higher ion influx resulted in an increased efficiency of light energy transformation, CO_2_ conductance, potential of light and dark reactions, and photosynthetic rate [133]. 

Phytohormones are the basic units of plant growth and development, and their interactions with EBL are important for improving the stress resistance mechanism [134]. Abscisic acid functions as a signaling molecule in response to drought stress, while its interaction with EBL regulates the expression of several genes, such as those involved in the biosynthesis of SOD, CAT, POD, and GR, which are involved in stress tolerance [135,136]. The results in Figure 3 show that EBL activates the enzymes SOD, CAT, and POD in banana chloroplasts. Thus, the active anti ROS defense system removed the free radicals, maintaining the relative stability of chloroplasts under drought stress. Moreover, ALA and EBL could directly remove a large amount of O^−^_2_, which can repair the membrane system of the chloroplast and protect it from senescence under stress conditions. In this regard, it was reported that exogenous application of EBL increased the activity of the phenylalanine ammonialyase (PAL) and POD enzymes [137], which increased the biosynthesis of phenolic compounds, ultimately leading to a reduced accumulation of H_2_O_2_ and O^−^_2_ content. It also improved the activity of UPD-glucose: flavonoid 3-*O*-glucosyltransferase (UFGT), the content of tannins and flavonoids, and the antioxidant capacity in grapevines [138].

Bananas are rich in phenols [139], which are one of the most important groups of secondary metabolites in plants [140] and have been shown to act as antioxidants that protect the cell structure and improve plant tolerance to stress conditions [141], in addition to their great influence on the sensory quality of the fruit (i.e., color, aroma, and flavor) [142]. The biosynthesis of ABA is important in promoting root growth and regulating shoot growth [143] via the regulation of transpiration (through stomatal closure) and photosynthesis [144]. The increased level of ABA also increased the activity of PAL [145]. The positive role of ABA on sugar content came from the accumulation of assimilates from the phloem into the fruit by strengthening the sink capacity [146]; however, it has been reported that the increase in ABA may be an indicator of the onset of plant senescence [147]. The reduction in bunch weight (Table 3) and fruit weight (Figure 4) under drought conditions could also be related to the increase in the ABA content, as it is negatively correlated with fruit weight, as reported in lemon fruit [148]. It is also involved in ethylene production, which in turn affects cellulase and polygalacturonase activity, leading to fruit softening and increased senescence processes [149]. Previous reports showed that EBL plays a role in the biosynthesis of ethylene [48], which plays a fundamental role in plant growth and development, especially under a variety of abiotic and biotic stresses. It significantly increased the transcription level of the ethylene signaling biosynthesis genes responsible for fruit ripening under stress conditions [150], which could explain the improved levels of TSS along with reduced levels of TA in “Williams” banana fruit (Figure 5). The increase in the fruit sugar content is usually associated with the increase in TSS, since sugar is the main component of TSS [151], although the increase in sugar contents was insignificant compared to the control (Figure 5). In this regard, it was found that the application of either ALA or EBL at a concentration of 30 mg·L^−1^ effectively reduced the content of ABA (Table 2) and, thus, could decrease ethylene production, protecting the chloroplast structure (Figure 2 and Table 1). This is so important to consider because the interactions among these phytohormones are critical, as higher concentrations of ALA or EBL could trigger higher ethylene production and, thus, improve the respiration rate and cell senescence, which could affect chloroplast efficiency and overall plant growth [33,42,48,133].

It can be deduced that the effective role of ALA or EBL on ‘Williams’ banana yield under drought stress is due to their promotion of photosynthesis as well as antioxidant enzyme activities, particularly those located mainly near the reaction center of PSI, which can scavenge O^−^_2_^.^, leading to an increase in apparent electron transport rates and the alleviation of photosynthetic photoinhibition [128,152]. The effect of ALA or EBL is concentration-dependent, and the highest values were measured in the plants treated with EBL (30 mg·L^−1^) followed by those treated with ALA (30 mg·L^−1^), while the lowest values were measured in the control plants. The concentration-dependent effect was also noticeable in the effective role of ALA (45 mg·L^−1^) on the fruit weight (Figure 4) and bunch weight (Table 3) of the stressed plants compared to the non-stressed ones received the same treatments. The same effect was noticed on the bunch weight with the application of EBL (15 mg·L^−1^) in both seasons (Table 3). It was reported that soil and foliar application of ALA or EBL significantly increased plant growth and productivity under stressful conditions [153,154]. These findings could justify the increase in the fruit length and diameter of the stressed plants compared to the non-stressed ones received the same treatments (Figure 4). The beneficial effect of ALA and EBL on improving the yield and fruit characteristics might also be related to their effective role under drought conditions in triggering ethylene biosynthesis, leading to the accumulation of glucose, fructose, and sucrose through the inhibition of pectinase and polygalacturonase [155]. In grapevines, BRs were found to increase soluble sugars by promoting the activity of invertase and sucrose synthase, as well as upregulating the expression of genes encoding invertase and mono- and di-saccharide transporters [156]. Glucose and fructose were largely responsible for active osmoregulation in moderately stressed plants [157]. Sugar accumulation was not the result of cell desiccation under stress conditions but was produced from carbon assimilation by non-stomatal photosynthesis [158] or from the plant reserve by active osmoregulation to maintain cell turgidity and minimize the damages caused by water stress [156]. However, these sugars were reported not to be fully utilized for fruit volume growth, even after irrigation was resumed [159], and this could also be a rationale for the reduction in fruit weight in most treated stressed plants compared to the non-stressed ones received the same treatments during both seasons (Figure 4). The accumulation of unutilized sugars in fruit cells could be the reason for increased TSS levels compared to the control in both seasons (Figure 5). The physiological role of EBL was more conspicuous than that of ALA at the same concentrations, and this could refer to the improved level of other endogenous BRs due to the exogenous application of EBL [119], compared to the very low level of natural ALA in the plant [33].

Overall, it could be suggested that both ALA and EBL improve the drought tolerance of ‘Williams’ banana through improving plant adaptation and/or avoidance mechanisms by improving nutrient and the CO_2_ uptake, osmotic adjustment, osmoprotection, antioxidation, and scavenging defense systems that determine the plant’s ability to produce yield under drought conditions [18]. It could also be stated that ALA (as a plant growth regulator) and EBL (as a phytohormone) are effective in very low concentrations, as previously reported [37,48,52]. Although the low concentrations like 15 and 30 mg·L^−1^ of ALA or EBL were found to be effective, the use of 45 mg·L^−1^ was also found effective on some parameters, basically those related to plant growth rather than fruit quality. This may open the door for future research on using higher concentrations, particularly under stress conditions.

## 4. Materials and Methods

### 4.1. Experiment

This research was carried out on the first and second ratoons of banana plants, *Musa acuminata*, subgroup Cavendish, cultivar Williams, in a private orchard located at El-Khatatba, Menoufia Governorate (30°21′60″ N, 30°49′60″ E, 28 m height above sea level), Egypt, during the 2018/2019 and 2019/2020 seasons. The weather data of the experimental site [160] are displayed in Table 4. Soil samples were randomly collected from the surface layer (0–30 cm) for physical and chemical analyses [161] and the data are displayed in Table 5.

The first and second ratoons of “Williams” banana mother plants, grown in sandy soil, were carefully inspected for the selection of 5th-leaf-stage seedlings similar in size and vigor and free from any disease symptoms. The seedlings were then planted at a 3 m × 3 m spacing during the first week of July of both seasons. They were grown under full sunlight, where the midday solar irradiance was about 1500–1700 µmol·m^−1^·s^−1^. The seedlings were divided into two groups; one group was subjected to the recommended crop water requirements (WR) used in the area (80 m^3^·plant^−1^·year^−1^), representing the control, whereas the other group was subjected to deficit irrigation at 75% WR (60 m^3^·plant^−1^·year^−1^). Two lateral lines of water pipes were set up on both sides of each plant row, with two drippers on each side of the plant; each dripper discharged 4 L·h^−1^. Reducing the discharge volume of each dripper to 3 L·h^−1^ reduced water amount to 75% WR.

At the 14th-leaf-stage of plant growth, plants were foliarly sprayed three times in biweekly intervals with liquid solutions of either 5-aminolevulinic acid hydrochloride, 97% ‘ALA’ (Sigma Aldrich, St. Louis, MO, USA) or 24-Epibrassinolide ‘EBL’ (Cosmo oil Co., Ltd., Tokyo, Japan) at concentrations of 15, 30, and 45 mg·L^−1^, supplemented with Tween 20 (0.1%) as a surfactant (Sigma Aldrich, St. Louis, MO, USA). The control plants were sprayed with distilled water at the same application times. The whole plant was sprayed until dripping. The plants selected for this experiment were subjected to other common agricultural practices of the entire orchard during both seasons. The experiment was laid out in a randomized complete block design (RCBD as split plots) of 14 treatments with 5 replicates each. Each replicate was represented by one plant. 

### 4.2. Leaf Analysis 

At the 20th-leaf-stage of plant growth (approximately two weeks after the third spray), the 3rd leaf from the top of the plant was collected for leaf analysis, as the evaluation of certain physiological indices related to plant growth and chloroplast degeneration under drought stress conditions. All used chemicals were imported from Sigma Aldrich, St. Louis, MO, USA. 

The leaf NPK content (mg·g^−1^ dw) was estimated according to the methodology of Wilde et al. [161]. Chloroplasts were extracted at 0–4 °C with a mixture of sucrose (100 mM), NaCl (200 mM), and K (50 mM) using phosphate buffer to maintain the pH at 7.4. The total chlorophyll content (mg·100 g^−1^ fw) was determined according to Wettstein [162]. The photosynthetic oxygen evolution of the chloroplasts (nmol·mg^−1^ fw) was measured according to a mass spectrometry method [163] using single quadrupole GC-MS (Thermo Fisher Scientific Inc., Waltham, MA, USA). Lipid peroxidation of the membrane was also determined with the MDA concentration (nmol·mg^−1^ fw) estimated at 532 nm using an extinction coefficient of 1555 mM^−1^·cm^−1^ and subtracting the absorbance at 600 nm for non-specific turbidity [164]. The chloroplast suspension (500 mL) was frozen in liquid N and stored at −20 °C for the determination of non-radical H_2_O_2_ (nmol·mg^−1^ fw) [165] and the EL percentage [166].

The extraction and evaluation of the activity of SOD (EC 1.15.1.1), CAT (EC 1.11.1.6), and POD (EC 1.11.1.7) (U·g^−1^·min^−1^ fw) were carried out according to the methods described by Beauchamp and Fridovich [167], Herzog and Fahimi [168], and Bergmeyer [169], respectively. Phytohormones (μg·g^−1^ fw), such as IAA and ABA, were determined according to Koshioka et al. [170] using high-performance liquid chromatography (M5 Micro flow HPLC system; SCIEX, Framingham, MA, USA), whereas CKs were determined using the methodology of Nicander et al. [171].

### 4.3. Yield and Fruit Quality

Banana bunches were harvested by late September–early October (15 months after planting) in both seasons, when fruit became fully developed (75% maturity ≈ 80–90 days after the opening of the first hand) [172]. Bunch weight (kg) was measured using a bench digital scale model 1200 MSP (Doran scales, Inc., Batavia, IL, USA). The number of hands per bunch, and the number of fruits (fingers) per hand were counted and averaged. Ten fruit per bunch were randomly selected to measure the fruit weight (g) using a bench-top digital scale model PC-500 (Doran scales, Inc., Batavia, IL, USA). The same ten fruits were used to measure and calculate the average fruit length (cm) and diameter (cm) using a digital caliper (Grizzly Industrial, Bellingham, WA, USA). The percentage of TSS was estimated at room temperature (≈22–23 °C) using a hand-held refractometer model RA-130 (KEM Kyoto Electronics Manufacturing Co., Ltd., Tokyo, Japan). Moreover, TA (g malic acid·100 g^−1^ pulp) and total sugars (g·100 g^−1^ pulp) were estimated as a percentage according to the AOAC protocol [173]. 

### 4.4. Statistical Analysis 

The data were first assessed for numerical normality and homogeneity of variance using the Shapiro–Wilk and Levene’s tests, respectively. The data were then statistically analyzed, and the analysis of variance (ANOVA) was performed using the SPSS software package (SPSS Inc., ver. 16, Chicago, IL, USA). The means were compared using the standard error values and a post-hoc Duncan’s multiple range test (DMRT) [174]. 

## 5. Conclusions

Under drought stress conditions, the application of ALA or EBL at a dosage of 30 mg·L^−1^ could be suggested to improve the stress resistance of banana plants. These two natural compounds effectively reduced plant oxidative stress by increasing antioxidant activity, thus maintaining chloroplast integrity and improving photosynthetic capacity, resulting in improved growth, development, and productivity. In the future, this study could incorporate the molecular basis of the banana plant defense mechanism to develop new drought-tolerant banana cultivars.

## Figures and Tables

**Table 1:** Effect of foliar spray with ALA or EBL on the malondialdehyde (MDA) content and electrolyte leakage (EL) of drought-stressed “Williams” banana plants during the 2018/2019 and 2019/2020 seasons (*n* = 5).

Foliar Spray	Water Requirements (WR)			
	2018/2019		2019/2020	
	100% WR	75% WR	100% WR	75% WR
MDA (nmol·mg^−1^ fw)				
Control	0.11 ± 0.040 a	0.17 ± 0.033 a	0.11 ± 0.013 a	0.19 ± 0.015 a
ALA 15 mg·L^−1^	0.09 ± 0.018 b	0.13 ± 0.024 b	0.11 ± 0.018 a	0.17 ± 0.023 ab
ALA 30 mg·L^−1^	0.05 ± 0.006 d	0.11 ± 0.005 c	0.05 ± 0.005 c	0.14 ± 0.080 c
ALA 45 mg·L^−1^	0.11 ± 0.028 a	0.17 ± 0.060 a	0.12 ± 0.014 a	0.19 ± 0.061 a
EBL 15 mg·L^−1^	0.05 ± 0.008 d	0.12 ± 0.004 b	0.07 ± 0.006 b	0.13 ± 0.029 c
EBL 30 mg·L^−1^	0.03 ± 0.004 e	0.08 ± 0.009 d	0.04 ± 0.004 d	0.09 ± 0.009 d
EBL 45 mg·L^−1^	0.07 ± 0.003 c	0.12 ± 0.017 b	0.07 ± 0.008 b	0.16 ± 0.018 b
EL (%)				
Control	54 ± 4.35 b	60.5 ± 4.71 a	57.5 ± 2.03 b	64 ± 2.13 b
ALA 15 mg·L^−1^	50 ± 5.11 b	62 ± 6.28 a	56 ± 1.90 b	61 ± 4.21 b
ALA 30 mg·L^−1^	42 ± 3.82 c	45 ± 5.04 bc	41 ± 2.41 c	46 ± 1.80 c
ALA 45 mg·L^−1^	62 ± 3.49 a	64 ± 6.00 a	67 ± 3.11 a	71 ± 3.65 a
EBL 15 mg·L^−1^	34 ± 4.11 d	43 ± 3.78 c	37 ± 2.08 d	42 ± 3.00 d
EBL 30 mg·L^−1^	26 ± 4.00 e	32 ± 5.26 d	27 ± 2.63 e	32 ± 2.18 e
EBL 45 mg·L^−1^	38 ± 3.99 d	48 ± 3.88 b	42 ± 1.98 c	47 ± 2.07 c

Means followed by the same letter within a column are not significantly different using DMRT (*p* ≤ 0.05).

**Table 2:** Effect of foliar spray with ALA or EBL on the auxin (IAA), cytokinins (CKs), and abscisic acid (ABA) contents in drought-stressed “Williams” banana plants during the 2018/2019 and 2019/2020 seasons (*n* = 5).

Foliar Spray	Water Requirements (WR)			
	2018/2019		2019/2020	
	100% WR	75% WR	100% WR	75% WR
IAA (μg·g^−1^ fw)				
Control	22.9 ± 0.78 e	20 ± 0.77 e	22.8 ± 0.8 e	19.7 ± 0.95 e
ALA 15 mg·L^−1^	26.1 ± 0.61 d	21.6 ± 1 d	25.9 ± 0.74 d	21.4 ± 1.11 d
ALA 30 mg·L^−1^	29.7 ± 89 b	27.8 ± 1.08 b	29.5 ± 1.4 b	27.6 ± 0.91 b
ALA 45 mg·L^−1^	26.8 ± 9 d	25.8 ± 0.92 c	26.5 ± 0.5 d	25.5 ± 0.88 c
EBL 15 mg·L^−1^	27.7 ± 55 c	24.5 ± 0.85 c	27.5 ± 0.67 c	24.3 ± 1.27 c
EBL 30 mg·L^−1^	32.2 ± 1.02 a	30.1 ± 1.2 a	32.1 ± 1.44 a	29.9 ± 1.25 a
EBL 45 mg·L^−1^	27.9 ± 0.87 c	27.3 ± 0.67 b	27.6 ± 0.6 c	27.0 ± 0.89 b
CKs (μg·g^−1^ fw)				
Control	7.0 ± 0.24 e	5.9 ± 0.33 e	6.9 ± 0.12 d	5.8 ± 0.37 e
ALA 15 mg·L^−1^	8.3 ± 0.35 d	7.4 ± 0.19 d	8.1 ± 0.17 c	7.3 ± 0.15 d
ALA 30 mg·L^−1^	9.4 ± 0.29 b	8.5 ± 0.31 b	9.2 ± 0.28 b	8.5 ± 0.19 b
ALA 45 mg·L^−1^	8.6 ± 0.33 d	7.4 ± 0.24 d	8.3 ± 0.35 c	7.5 ± 0.34 c
EBL 15 mg·L^−1^	8.9 ± 0.4 d	8.0 ± 0.36 c	8.6 ± 0.41 c	7.7 ± 0.49 c
EBL 30 mg·L^−1^	10.3 ± 0.42 a	9.0 ± 0.51 a	9.9 ± 0.48 a	8.9 ± 0.17 a
EBL 45 mg·L^−1^	9.0 ± 0.56 c	8.3 ± 0.39 b	8.9 ± 0.4 b	7.9 ± 0.23 c
ABA (μg·g^−1^ fw)				
Control	0.61 ± 0.09 a	0.75 ± 0.07 a	0.64 ± 0.02 a	0.77 ± 0.03 a
ALA 15 mg·L^−1^	0.57 ± 0.06 a	0.66 ± 0.04 b	0.59 ± 0.02 b	0.69 ± 0.06 b
ALA 30 mg·L^−1^	0.52 ± 0.04 b	0.55 ± 0.08 c	0.55 ± 0.04 b	0.59 ± 0.03 c
ALA 45 mg·L^−1^	0.54 ± 0.09 b	0.63 ± 0.05 b	0.58 ± 0.05 b	0.67 ± 0.04 b
EBL 15 mg·L^−1^	0.53 ± 0.03 b	0.61 ± 0.04 b	0.56 ± 0.04 b	0.63 ± 0.03 b
EBL 30 mg·L^−1^	0.48 ± 0.04 c	0.51 ± 0.03 d	0.50 ± 0.03 c	0.53 ± 0.02 d
EBL 45 mg·L^−1^	0.51 ± 0.04 b	0.59 ± 0.03 c	0.54 ± 0.03 b	0.61 ± 0.05 c

Means followed by the same letter within a column are not significantly different using DMRT (*p* ≤ 0.05).

**Table 3:** Effect of foliar spray with ALA or EBL on the bunch weight, number of hands per bunch, and number of fruits per hand of drought-stressed “Williams” banana plants during the 2018/2019 and 2019/2020 seasons (*n* = 5).

Foliar Spray	Water Requirements (WR)			
	2018/2019		2019/2020	
	100% WR	75% WR	100% WR	75% WR
Bunch weight (kg)				
Control	28.8 ± 0.7 f	25.2 ± 0.88 c	28.0 ± 0.4 e	24.6 ± 0.67 d
ALA 15 mg·L^−1^	33.6 ± 0.47 c	31.6 ± 0.3 b	29.8 ± 0.7 e	32.4 ± 0.51 c
ALA 30 mg·L^−1^	34.4 ± 1 b	32.0 ± 0.41 b	34.5 ± 1.2 a	34.8 ± 0.4 ab
ALA 45 mg·L^−1^	31.2 ± 0.5 e	32.5 ± 0.5 b	31.4 ± 0.3 c	32.5 ± 0.71 c
EBL 15 mg·L^−1^	31.6 ± 0.25 e	34.5 ± 0.32 a	30.2 ± 0.65 d	34.0 ± 0.39 b
EBL 30 mg·L^−1^	36.0 ± 0.7 a	35.2 ± 0.4 a	34.4 ± 0.81 a	35.5 ± 0.53 a
EBL 45 mg·L^−1^	32.2 ± 0.39 d	32.8 ± 1.03 b	32.5 ± 0.36 b	32.5 ± 0.37 c
Number of hands·bunch^−1^				
Control	10.6 ± 0.33 d	10.0 ± 0.29 d	10.3 ± 0.19 d	9.5 ± 0.34 d
ALA 15 mg·L^−1^	11.5 ± 0.28 c	11.4 ± 0.36 c	11.0 ± 1.0 c	11.0 ± 0.7 c
ALA 30 mg·L^−1^	12.5 ± 0.31 b	12.0 ± 0.22 b	12.2 ± 0.78 b	11.8 ± 0.42 b
ALA 45 mg·L^−1^	11.8 ± 0.4 c	11.2 ± 0.3 c	11.6 ± 0.27 c	11.0 ± 0.35 c
EBL 15 mg·L^−1^	12.5 ± 0.72 b	12.0 ± 0.48 b	12.2 ± 1.03 b	11.8 ± 0.47 b
EBL 30 mg·L^−1^	14.0 ± 0.85 a	13.5 ± 0.27 a	13.8 ± 0.061 a	13.2 ± 0.401 a
EBL 45 mg·L^−1^	12.2 ± 0.78 b	12.0 ± 0.38 b	12.5 ± 0.23 b	11.6 ± 0.17 b
Number of fruits·hand^−1^				
Control	15.5 ± 0.25 e	14.1 ± 0.12 d	15.2 ± 0.21 e	13.7 ± 0.27 d
ALA 15 mg·L^−1^	16.0 ± 0.21 d	15.3 ± 0.09 c	15.8 ± 0.32 d	15.0 ± 0.28 c
ALA 30 mg·L^−1^	17.2 ± 0.2 b	16.5 ± 0.3 b	16.8 ± 0.19 b	16.5 ± 0.31 a
ALA 45 mg·L^−1^	16.5 ± 0.42 c	16.2 ± 0.19 b	15.2 ± 0.24 e	16.0 ± 0.19 b
EBL 15 mg·L^−1^	16.8 ± 0.31 c	16.1 ± 0.22 b	16.2 ± 0.19 c	16.0 ± 0.8 b
EBL 30 mg·L^−1^	17.9 ± 0.27 a	17.4 ± 0.1 a	17.8 ± 0.5 a	16.8 ± 0.24 a
EBL 45 mg·L^−1^	16.6 ± 0.15 c	16.2 ± 0.09 b	16.0 ± 0.23 c	15.8 ± 0.41 b

Means followed by the same letter within a column are not significantly different using DMRT (*p* ≤ 0.05).

**Table 4:** Weather data of El-Khatatba, Menoufia, Egypt as the average of the 2018, 2019, and 2020 seasons.

	Temperature	Humidity	Rainfall	Wind Speed	Cloud	Sun	UV Index
	(°C)	(%)	(mm·monh^−1^)	(km·h^−1^)	(%)	(days·month^−1^)	
Winter	17.0	56.0	4.7	14.0	22.0	24.1	4.9
Spring	25.6	44.9	3.3	15.6	13.8	27.7	6.8
Summer	32.9	50.7	0.0	14.5	5.1	30.7	8.0
Fall	27.2	56.8	2.3	13.0	11.4	28.8	6.7

**Table 5:** Soil analysis of the experimental site at El-Khatatba, Menoufia, Egypt as the average of the 2018/2019 and 2019/2020 seasons.

Characteristics	Soil Depth (0–30 cm)
Texture	Sandy
Coarse + fine sand (%)	89.7
Silt (%)	6.0
Clay (%)	4.3
Organic matter (%)	0.65
N (%)	0.88
P (%)	0.25
K (%)	0.41
CaCO_3_ (%)	1.65
HCO_3_^−^ (meq·L^−1^) (1:20 extract)	2.1
CO_3_^2−^ (meq·L^−1^) (1:20 extract)	0.0
Cl^−^ (meq·L^−1^) (1:20 extract)	2.2
SO_4_^2−^ (meq·L^−1^) (1:20 extract)	2.8
Ca^2+^ (meq·L^−1^) (1:20 extract)	2.8
Mg^2+^ (meq·L^−1^) (1:20 extract)	1.1
Na^+^ (meq·L^−1^) (1:20 extract)	0.5
K^+^ (meq·L^−1^) (1:20 extract)	1.6
Fe (mg·L^−1^)	2.5
Mn (mg·L^−1^)	1.58
Zn (mg·L^−1^)	0.23
EC (ds·m^−1^) (1:5 extract)	4.5
pH (1:5 extract)	8.1
Bulk density (g·cm^−3^)	1.55
Field capacity	13.7
Wilting point	4.8

## Data Availability

Not applicable.
